# Exploring Tumor Immune Microenvironment and Its Associations With Molecular Characteristics in Melanoma

**DOI:** 10.3389/fonc.2022.821578

**Published:** 2022-04-21

**Authors:** Jiangyuan Wang, Cong Peng, Wentao Dai, Xiang Chen, Jing Meng, Taijiao Jiang

**Affiliations:** ^1^ Institute of Systems Medicine, Chinese Academy of Medical Sciences & Peking Union Medical College, Beijing, China; ^2^ Suzhou Institute of Systems Medicine, Suzhou, China; ^3^ Department of Dermatology, Xiangya Hospital, Central South University, Changsha, Hunan, China; ^4^ Hunan Key Laboratory of Skin Cancer and Psoriasis, Xiangya Hospital, Central South University, Changsha, China; ^5^ NHC Key Lab of Reproduction Regulation (Shanghai Institute for Biomedical and Pharmaceutical Technologies) & Shanghai Engineering Research Center of Pharmaceutical Translation, Fudan University, Shanghai, China; ^6^ Shanghai Key Laboratory of Gastric Neoplasms, Department of General Surgery, Shanghai Institute of Digestive Surgery, Ruijin Hospital, Shanghai Jiao Tong University School of Medicine, Shanghai, China; ^7^ Institute of Basic Medical Sciences Chinese Academy of Medical Sciences, School of Basic Medicine Peking Union Medical College, Beijing, China; ^8^ Guangzhou Laboratory, Guangzhou, China

**Keywords:** melanoma, tumor microenvironment, molecular characteristics, survival outcome, chemokines and their receptors

## Abstract

**Background:**

The tumor microenvironment (TME), which involves infiltration of multiple immune cells into the tumor tissues, plays an essential role in clinical benefit to therapy. The chemokines and their receptors influence migration and functions of both tumor and immune cells. Also, molecular characteristics are associated with the efficacy of melanoma therapy. However, there lacked exploration of immune characteristics and the association with molecular characteristics.

**Methods:**

We collected the currently available 569 melanoma samples that had both the genomic and transcriptional data from TCGA and SRA databases. We first identified TME subtypes based on the developed immune signatures, and then divided the samples into two immune cohorts based on the immune score. Next, we estimated the compositions of the immune cells of the two cohorts, and performed differential expression genes (DEGs) and functional enrichments. In addition, we investigated the interactions of chemokines and their receptors under immune cells. Finally, we explored the genomic characteristics under different immune subtypes.

**Results:**

TME type D had a better prognosis among the four subtypes. The high-immunity cohort had significantly high 16 immune cells. The 63 upregulated and 384 downregulated genes in the high-immunity cohort were enriched in immune-related biological processes, and keratin, pigmentation and epithelial cells, respectively. The correlations of chemokines and their receptors with immune cell infiltration, such as CCR5-CCL4/CCL5 and CXCR3-CXCL9/CXCL10/CXCL11/CXCL13 axis, showed that the recruitments of 11 immune cells, such as CD4T cells and CD8T cells, were modulated by chemokines and their receptors. The proportions of the four TME subtypes in each molecular subtype were comparable. The two driver genes, CDKN2A and PRB2, had significantly different MAFs between the high-immunity and low-immunity.

**Conclusion:**

We dissected the characteristics of immune infiltration, the interactions of chemokines and their receptors under immune cells, and the correlation of molecular and immune characteristics. Our work will enable the reasonable selection of anti-melanoma treatments and accelerate the development of new therapeutic strategies for melanoma.

## Introduction

Melanoma is the most deadly kind of skin cancer in which cancer cells form in melanocytes (1). It is also a highly heterogeneous cancer, which has been putting a heavy burden on human health with a higher risk of metastasis and a high death rate (2). The number of cases of melanoma has been increasing over the past 30 years. It was reported that there were more than 324,600 diagnosed cases and 57,000 deaths worldwide in 2020 (2).

The tumor microenvironment (TME), which involves infiltration of multiple immune cells into the tumor tissues, plays an essential role in prognosis and clinical benefit to therapy. Some studies have reported the infiltration of immune cells in the melanoma microenvironment (3, 4). Immune cells play a dualistic role, including promoting tumor development or anti-tumor development, and they create different microenvironments depending on their types and interactions (5). The chemokines and their receptors influence migration and functions of both tumor and immune cells, which significantly impacts tumor fate and is critical in melanoma control and progression (6). Therefore, it is important to understand the characteristics of immune infiltration and the interactions of chemokines and their receptors under immune cells.

A study reported a widely suitable immune subtyping method, which identified six immune subtypes among more than 10,000 tumors comprising 33 diverse cancer types, based on the five representative signatures including IFN-r, TGF-b, Macrophage, Lymphocyte and Wound healing based on 160 immune expression signatures (7). Then, a pan-cancer study extended to 29 functional gene expression signatures (Fges) for immune subtyping (8). It selected Fges representing the major functional components and immune, stromal, and other cellular populations of the tumor by integrating knowledge from multiple publications. Then, the ‘29 Fges’ were chosen by using tSNE projections and Mann-Whitney tests, and were divided into four groups according to their functions, processes or cell types. Finally, the tumor samples were classified into four subtypes using unsupervised clustering based on the ssGSEA scores of the expression patterns of 29 Fges. The two studies focused on the pan-cancer, but lacked exploration of immune characteristics for melanoma. A recent study revealed three immune cell infiltration (ICI) clusters associated with different immune subtypes, and assessed the predictive values of the ICI scores for the immune therapy benefit in melanoma (4). The ICI clusters were identified using limited features, and this study provided little information for the association of TME and molecular characteristics for melanoma.

The Cancer Genome Atlas (TCGA) developed a molecular subtyping method based on the mutations for melanoma (9). The resulting four molecular subtypes were BRAF subtype characterized by BRAF hot-spot mutations, RAS subtype defined by the presence of RAS hot-spot mutations in all three RAS family members (N/K/HRAS), NF1 subtype featured by NF1 loss-of-function mutations, and Triple-Wild-Type defined by the absence of hot-spots BRAF, N/H/K/-RAS or NF1mutations. A study showed the whole genome mutation landscape of 183 samples of three melanoma (cutaneous, acral and mucosal), and revealed diverse carcinogenic processes across the three subtypes (10). These two studies depicted molecular characteristics only.

To fill in the gaps of these previous studies, we employed the currently available 569 melanoma samples that had both the genomic and transcriptional data, and we took the advantages of the developed immune and molecular subtyping methods to dissect the characteristics of immune infiltration, the chemokine-receptor pairs under immune cells, and the correlation of molecular and immune characteristics. We identified candidate biomarkers that can be used to predict clinical benefits for melanoma, which enables the reasonable selection of anti-melanoma treatments. Our work contributes to the understanding of melanoma immune microenvironment and the association with molecular characteristics, which will facilitate the development of new therapeutic strategies for melanoma.

## Materials and Methods

### Melanoma Data Resources

We collected 569 melanoma samples from four datasets from the Cancer Genome Atlas (TCGA, https://portal.gdc.cancer.gov/) and SRA databases, of which 567 samples had both transcriptional and genomic data and 2 samples had the transcriptional data only (**Supplementary Table S1**). Accessions for these 569 samples are the TCGA skin cutaneous melanoma project (TCGA-SKCM), phs001036 (11), phs000452 (12) and SRP070710 (13).

### RNA-Seq Processing

99 of the 569 samples had no normalized counts, so we aligned their RNA-seq reads to the human reference genome GRCh37 using STAR (14) v2.7.5b with default parameters, and quantified the resulting gene expression count matrix with RSEM (15) v1.3.1. Then we integrated with the count matrix of the 470 samples, and did quantile normalization. The batch effect was eliminated by Combat_seq (16).

### Detection of CNV

The CNVs of the 469 samples were predicted by GISTIC (17) with 0.95 GISTIC significance threshold. The CNV levels were extracted from the output files for all genes using a cutoff of ±2. The CNVs of the 98 samples were downloaded from cBioportal (https://www.cbioportal.org/datasets).

### TME Subtyping

29 Fges representing four immune signature modules, including anti-tumor microenvironment, pro-tumor microenvironment, angiogenesis fibrosis and malignant cell properties, were selected to identify TME subtypes. The detailed information about the 29 Fges was showed in **Supplementary Table S2**. 29 Fges signature scores were calculated using Single-sample gene set enrichment analysis (ssGSEA) in python3 and median-scaled for all the samples. Then we classified the samples using louvain community detection algorithm to identify immune subtypes based on the signature scores. The immune and stromal cell scores were estimated by the ESTIMATE method (18). According to the immune scores, we divided the patients into two cohorts, high-immunity and low-immunity for the following analysis.

### Evaluation of Overall Survival

To evaluate the impact of immune features on patients’ outcome, the R package survival and survminer were used to analyze the Overall Survival (OS) using the Kaplan-Meier method. P-value of less than 0.05 was considered to be statistically significant.

### Depiction of Immune Cell Population and the Relationship With Chemokines and Their Receptors

To identify the differences of the fractions of immune cell types between the high-immunity and low-immunity cohorts, we used ImmuneCellAI (19) to estimate the compositions of the immune cells. For each immune cell, the statistical difference in the compositions between the high-immunity and low-immunity cohorts was evaluated by Mann-Whitney U test. The relationships of immune cells and the interaction between chemokines and their receptors were evaluated by Spearman’s correlation. Abs(r) > 0.5 and p < 0.05 were considered to be related and statistically significant, respectively. The interaction network of Chemokines and their receptors for immune infiltration in melanoma was constructed by Gephi v0.9.2.

### Detection of Significantly Mutated Genes and Their CNVs

To identify driver genes, MutSigCV (20) was used based on 568 samples. Genes with q < 0.1 were deemed significant, and the genes whose MAFs were less than 1% were removed. The statistical differences of the identified driver genes between the high-immunity and low-immunity cohorts were performed by Fisher’s exact test. The distributions of the driver genes in the high-immunity and low-immunity cohorts were performed by R package Maftools (21) and ComplexHeatmap (22). For the CNVs of these significantly mutated genes, the statistical differences between the high-immunity and low-immunity cohorts were evaluated by Fisher’s exact test.

### Identification of Differentially Expressed Genes and Functional Enrichment Analysis

The differential gene expression analyses between the high-immunity and the low-immunity cohorts were performed by DESeq2 (23), and the estimated significance level (P-value) for the differential expression analysis was adjusted by Benjamini and Hochberg False Discovery Rate (FDR) correction. The Gene Ontology (GO) enrichment analyses of differential expression genes were performed by the R package clusterProfiler (24, 25).

### Molecular Subtyping

We classified the samples into four molecular subtypes: BRAF subtype characterized by BRAF hot-spot mutations, RAS subtype defined by the presence of RAS hot-spot mutations in the three RAS families (N-, K-, and H-RAS), NF1 subtype characterized by the NF1 loss-of-function mutations, and Triple-Wild-Type defined by the absence of hot-spots BRAF, N/H/K/-RAS, or NF1mutations. Hot-spot mutations with p-values less than 1e-8 for the genes were chosen from HotSpotsAnnotations.

## Results

### Different Survival Outcomes of the TME Subtypes in Melanoma

**Figure 1:**
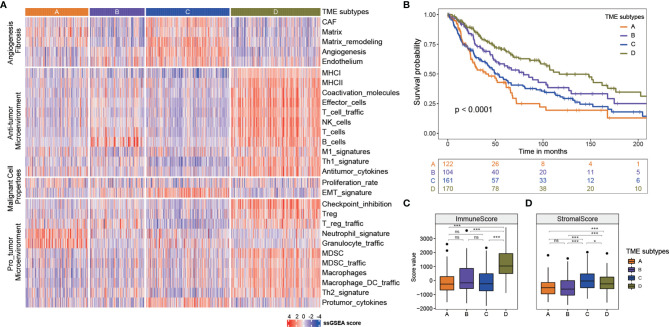
Four TME subtypes identified in melanoma. **(A)** Four TME subtypes showed differences in expression patterns of these 29 Fges. **(B)** Kaplan-meier curves of overall survival for four TME sbutypes. Log rank test p <0.0001. The mouths were calculated by years*365/12 or days/365*12. **(C)** The immune score of four TME subtypes. *** p<0.001; ns, no significance. **(D)** The stromal score of four TME subtypes. * p<0.05; *** p<0.001; ns, no significance.

To understand how the interaction of the tumor cells and immune and stromal cells influences the prognosis, we did the TME subtyping and the prognostic analysis of the 569 melanoma samples. The 29 Fges constitute four groups, including anti-tumor, pro-tumor microenvironment, angiogenesis fibrosis and malignant cell properties (8). The expression patterns of these 29 Fges were used to identify the immune subtype. The 569 samples were classified into four immune subtypes (**Figure 1A**). Compared with the other immune subtypes, TME type D had higher expressions in genes involved in anti-tumor immune and tumor-promoting processes. TME types A and C had higher expressions in genes involved in angiogenesis fibrosis. Furthermore, TME type B displayed higher gene expression levels in B_cells Fges than types A and C, and TME type A had the highest gene expression levels in neutrophil_signature and granulocyte_traffic.

To assess the prognostic values of TME subtypes, the Kaplan-Meier method was used to analyze the overall survival (the log-rank test, A: B: C: D, *p* < 0.0001, **Figure 1B**). The overall survival analysis showed that TME subtype D was characterized by a better overall survival, whereas TME subtypes A and C had the worse prognosis. TME subtype B had an intermediate survival outcome. Then, the stromal score and immune score of these four TME subtypes were calculated by ESTIMATE algorithm to measure the presence of tumor-associated stroma and the immune score. As shown in **Figure 1C**, the immune score of TME subtype D was significantly higher than that of the other subtypes, indicating subtype D had an immune-hot phenotype with a better prognosis (Mann–Whitney *U* test, *p* < 0.05). In terms of the stromal score, subtype C represented a higher score, while the subtype B had the lowest score (Mann–Whitney *U* test, *p* < 0.05, **Figure 1D**).

### Differences of the Immune Cells Population Between Immune Subtypes

**Figure 2:**
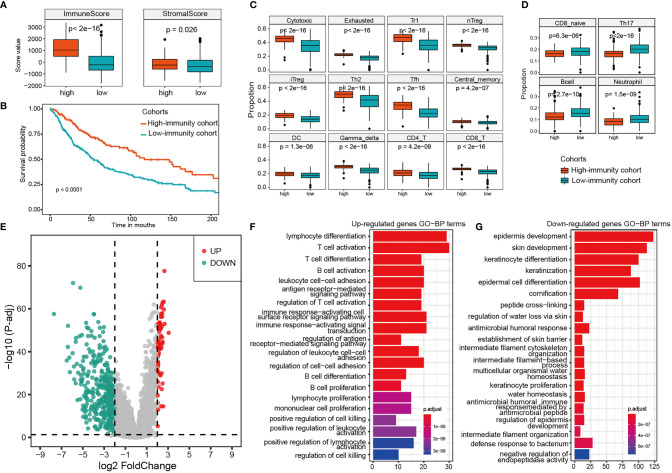
Differences of the immune cells population, expression analysis and functional enrichments between the high-immunity and the low-immunity cohort. **(A)** The immune score and stromal score of the high-immunity and the low-immunity cohort. **(B)** Kaplan-meier curves of overall survival for the two immunity cohorts. Log rank test, p <0.0001. **(C)** 16 significantly higher immune cells in the high-immunity cohort. **(D)** 4 significantly higher immune cells in the low-immunity cohort. **(E)** Volcano Plot of the DEGs for the high-immunity cohort. **(F, G)** GO function enrichments analysis of up-regulated and down-regulated genes for the high-immunity cohort.

Tumor-infiltrating immune cells are able to deeply influence tumor progression and patient outcome. So based on the immune score, we divided the samples into two cohorts, a high-immunity cohort including 175 samples from TME subtype D, and a low-immunity cohort including 393 samples from TME subtypes A, B and C (**Figures 2A, B**). The overall survival analysis showed that the high-immunity cohort had a better prognosis, whereas the low-immunity had a worse outcome (**Figure 2B**). Furthermore, ImmuneCellAI method was employed to measure the immune cell compositions. As shown in **Figures 2C, D**, the compositions of the immune cells varied between the two cohorts. The high-immunity cohort had significantly higher cytotoxic T cells, exhausted T cells, T regulatory type 1 (Tr1) cells, natural regulatory T (nTreg) cells and induced regulatory T (iTreg) cells, T helper type 2 (Th2) cells, T follicular helper (Tfh) cells, central memory T (TCM) cells, as well as dendritic cells (DC), gamma delta T cells and T lymphocytes (both CD4 T cell and CD8 T cell) (Mann-Whitney U test, p < 0.001, **Figure 2C**), while the low-immunity had significantly higher naive CD8 T cells, helper 17 (Th17) cells, B cells and neutrophil (Mann-Whitney U test, p < 0.001, **Figure 2D**). On the contrary, macrophage, mucosal associated invariant T (MAIT) cells, natural killer (NK) cells showed decreased differences between the two cohorts (Mann-Whitney U test, 0.001< p < 0.05), and there were no statistically significant differences in naive CD4 T cells, T helper type 1 (Th1) cells, effector memory T (TEM) cells, natural killer T (NKT) cells and monocyte cells between the two cohorts (**Supplementary Figure S1**).

### Differential Expression Analysis and Functional Enrichments Between Immune Subtypes

The differential expression analysis between the high-immunity and low-immunity cohorts was performed based on the expression profiles of the 569 melanoma samples. The genes with the FDR < 0.05 and abs (log2 FoldChange) > 2 were selected as differentially expressed between the two cohorts. As a result, there were 63 upregulated and 384 downregulated genes in the high-immunity cohort (**Figure 2E**). PLA2G2D, IFNG, CD8B and NKG7 were found to be significantly upregulated, which are associated with tumor immunity (**Supplementary Table S3**). LAG3 is also significantly upregulated, which has primarily been shown to inhibit activity of T-cells (26). However, it was reported that LAG3 expression was associated with an improved overall survival (OS) compared to patients without LAG3 expression in oesophageal adenocarcinoma (27). LAG3 may serve as a biomarker for a strong immune response, and the two opposite immune functions (positively or negatively) of LAG3 can be attributed to the complexity of tumor immune microenvironment and tumor heterogeneity (28). The function of LAG3 in melanoma needs further experiments to verify. The downregulated genes were mainly keratin, pigmentation, and epithelial genes such as KLK7 and IVL (**Supplementary Table S3**).

Furthermore, functional enrichments by clusterProfiler found that the upregulated genes in high-immunity cohort were enriched in immune-related biological processes, such as T cell activation, cell killing and leukocyte cell−cell adhesion (**Figure 2F**), indicating a favorable lymphocyte activation related to the immune system. Some upregulated genes, such as ADORA2A (29) and SLAMF6 (30), have been experimentally verified to be associated with tumor infiltration and T cell activation. In addition, TIGIT, which is a T-cell immune receptor with Ig and ITIM domains (31) and was upregulated in the high-immunity, has been recently identified as an attractive cancer immunotherapy target, because of its key role in interactions between the cells within the tumor microenvironment. The down-regulated genes were enriched in cornification, epidermis development, and keratin-related process (**Figure 2G**), all of which are associated with forming the outermost skin barrier (32). For example, human tissue kallikreins 7 (hKLK7) takes part in skin desquamation, which has been involved in keratinocyte cell shedding to catalyze the degradation of intercellular cohesive structures at the skin surface (33).

### Different Chemokine–Receptor Pairs in Immune Cells

**Figure 3:**
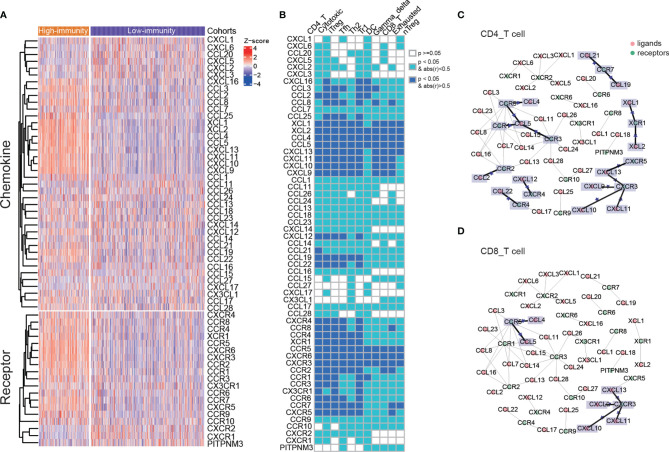
Different expressions of chemokines and their receptors between two immunity cohorts, and the chemokine–receptor pairs in immune cells. **(A)** The differences in gene expressions of the chemokines and their receptors between the high-immunity and low-immunity cohort. **(B)** The correlations of chemokines and their receptors with immune cell infiltration. **(C, D)** Chemokine/receptor networks for immune infiltration of CD 4 T cells and CD 8 T cells. For a pair of interacting chemokine and receptor genes, if both were significantly correlated with immune cell infiltration, a star was placed on the edge connecting the chemokine and receptor. Statistical significance was calculated using Spearman’s correlation.

Patient prognosis is related to the variety of immune cells. Particular chemokine and their receptors that are expressed on tumor and immune cells are strongly associated with patient prognosis as well. The immune cells are recruited from the circulation to the tumor microenvironment through the chemokine–receptor pairs, all of which play the essential roles in cell migration, proliferation and survival (34). We examined the differences in gene expressions of the chemokines and their receptors between the high-immunity and low-immunity cohort. The chemokines, XCL1, XCL2, CCL4, CCL5, CXCL13, CXCL11, CXCL10 and CXCL9 were found to be upregulated in the high-immunity cohort, which recruit different subsets of immune cells into the tumor microenvironment. Most of the chemokine receptors were overexpressed in the high-immunity cohort as well (**Figure 3A**).

The chemokine–receptor pairs with immune cell infiltration showed that the recruitments of CD4 T cells, cytotoxic T cells, iTreg, Tfh, Th2, Tr1 cells, DC cells, gamma delta T cells, CD8 T cells, exhausted T cells and nTreg cells were modulated by chemokines and their receptors (**Supplementary Figures 2A, B**). The latter 5 cells had less significantly related chemokine receptors (Spearman’s correlation, abs(r) > 0.5, p < 0.05, **Figure 3B**). The chemokine–receptor pairs in all of the above immune cells were displayed in **Supplementary Table S4**. CCR5-CCL4/CCL5 and CXCR3-CXCL9/CXCL10/CXCL11/CXCL13 axis were significantly correlated with CD 4 T and CD 8 T cells, indicating that these chemokine–receptor pairs may recruit the T lymphocytes into tumor (**Figures 3C, D**). The role for CCR5 has been documented in T-cell recruitment to the tumor site, and local production of CCL5 or CCL3 induces selective recruitment of CD8 T cells and CTL-dependent tumor suppression in mouse models (35). It showed that high levels of CXCL9 were associated with strong infiltration of malignant melanoma by CD8 T cells and improvement in patient survival (36). In addition, CD 4 T cell was significantly associated with other chemokine–receptor pairs, including XCR1-XCL1/XCL2, CCR7-CCL19/CCL21, CCR3/CCR1-CCL5, CCR2-CCL2, CXCR4-CXCL12 and CCR4-CCL22 (**Figure 3C**). Overexpressed chemokines and their receptors were likely to be involved in the anti-tumor immune response by promoting the migration of immune cells.

### The Genomic Characteristics Under Different Immune Subtypes

**Figure 4:**
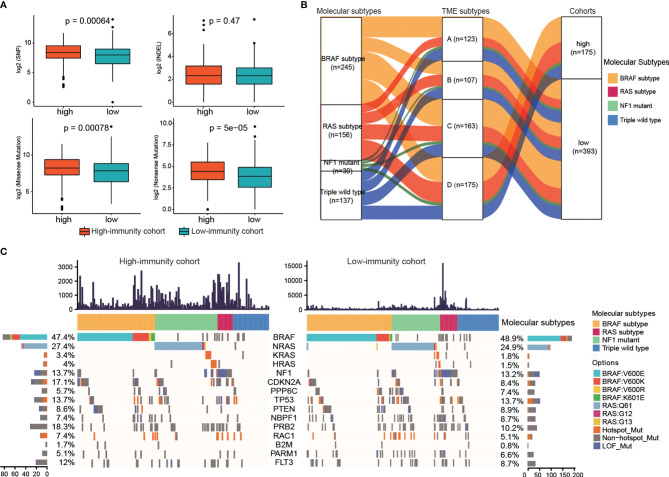
The genomic characteristics under different immune subtypes. **(A)** The differences in the number of somatic mutations between the two immunity cohorts. **(B)** Alluvial diagram of molecular subtypes distribution under TME subtypes and two immunity cohorts. **(C)** Waterfall plot of the frequency distributions of the significantly mutated genes. The numbers of the mutations of each sample were showed in upper panel.

The differences of somatic mutations, including single nucleotide polymorphism (SNP), insertion (INS), deletion (DEL), missense mutations and nonsense mutations, between the high-immunity and low-immunity cohort were investigated. The high-immunity cohort held a significantly larger number of SNP, missense and nonsense mutations than that in the low-immunity (Mann-Whitney U test, p < 0.05, **Figure 4A**). Then, we identified the significantly mutated genes, which were CDKN2A, NRAS, PPP6C, BRAF and TP53, PTEN, NBPF1, NF1, as well as PRB2, RAC1, B2M, PARM1, FLT3. Considering that HRAS and KRAS belong to the RAS family, the two genes were thought to be significantly mutated genes (9). We observed that two genes (CDKN2A and PRB2) had different MAFs between the high-immunity and low-immunity cohort (Fisher’s exact test, p < 0.05, **Supplementary Table S5**). The CDKN2A and PTEN genes were significantly deleted in the low-immunity cohort, whereas RAC1 was significantly amplified in the low-immunity cohort (Fisher’s exact test, p < 0.05, **Supplementary Table S5**).

Furthermore, to explore the relationship of the immune subtypes and the molecular subtypes, we did molecular subtyping based on the mutations (9). As shown in **Figure 4B**, the alluvial diagram displayed the distribution of the TME subtypes under different molecular subtypes and immune-cohorts. The proportions of the four TME subtypes in each molecular subtype were different. BRAF subtype showed the largest representations in all the four TME subtypes, while NF1 mutant subtype displayed the smallest representations (**Supplementary Figures S3A, B**). The proportion of RAS subtype in TME subtype A was lower than that in Triple wild type (20.33% for RAS-subtype, 26.83% for Triple wild type), while the proportions of RAS subtype in the other three TME subtypes were higher than that in Triple wild type (29.91%, 25.77%, 32.57% for RAS-subtype in TME B, C and D, and 28.04%, 22.09%, 21.71% for Triple wild type in TME B, C and D).

The MAFs of the 15 significantly mutated genes, of which 5 genes were used to do molecular subtyping, in the high-immunity and low-immunity cohorts were illustrated in **Figure 4C**. The BRAF subtype represented the largest molecular classification, which constituted 47.4% and 48.9% of the high-immunity cohort and the low-immunity cohort, respectively. The most frequent BRAF mutation targeted the V600 amino acid residue. The second largest subtype was RAS family members (NRAS, 27.4% of the high-immunity cohort and 24.9% of the low-immunity; KRAS, 3.4% and 1.8% of the high-immunity cohort and the low-immunity cohort; HRAS, 4% and 1.5% of the high-immunity cohort and the low-immunity cohort). The RAS subtype included G12, G13 and Q61 hot-spots mutations without the BRAF V600 and K601 mutations. The NF1 subtype contributed to 13.7% and 13.2% of the high-immunity and low-immunity cohort, respectively. NF1 is a significantly mutated gene in the MAPK pathway, which downregulates RAS activity as a negative regulator of the RAS signal transduction pathway (37). The Triple-WT subtype was defined by a lack of hot-spot BRAF, N/H/K-RAS or NF1 mutations.

## Discussion

Due to its high mutational load, melanoma represents one of the most immunogenic tumors with the potential to immune responses. To understand the immune microenvironment and molecular characteristics, we analyzed the transcriptional and genomic data of 569 melanoma samples from public databases. We identified four immune microenvironment subtypes. TME subtype D represented the overexpression of anti-tumor microenvironment, indicating that there were more immune features in this subtype. The TME subtypes A and C had the higher expressions of angiogenesis fibrosis comprising major stromal components. When it came to the prognostic benefit, TME subtype D had a better outcome, while TME subtype A represented a worse overall survival. Furthermore, TME subtype D had a significantly higher immune score than the other subtypes. The samples were divided into a high-immunity cohort and a low-immunity cohort based on their immune scores.

The high-immunity cohort had significantly higher T lymphocytes (CD 4 T and CD 8 T cells) and others immune cells such as Tr1, Th2, nTreg and iTreg, which explained the better overall survival of the high-immunity cohort. The differential expression analysis between the high-immunity and low-immunity cohorts showed that the high-immunity cohort had 63 upregulated and 384 downregulated genes. The 63 upregulated genes, including PLA2G2D, IFNG, LAG3, CD8B and NKG7, were associated with tumor immunity. The downregulated genes, such as KLK7 and IVL, were mainly associated with keratin, pigmentation, and epithelial genes. IVL may play an important role in the progression of melanoma (38). The upregulated genes were enriched in canonical immune biological processes, such as T cell activation and cell killing. As expected, the downregulated genes were enriched in cornification, epidermis development, and keratin-related process, all of which are likely able to modulate the tumor-development-related pathways (33).

Chemokines are a kind of chemotactic cytokines, which recruit lymphocytes through specific binding with their receptors in a specific way, and play an important role in the process of human inflammatory response and immune regulation (39). Chemokine receptors take part in the tumor proliferation, differentiation, invasion, and metastasis biological processes (34, 39). We found that the chemokines and their receptors, including XCL1, XCL2, CCL4, CCL5, CXCL13, CXCL11, CXCL10, CXCL9 and CCR5, CXCR3, CXCR6, significantly upregulated in the high-immunity cohort. Furthermore, CCR5-CCL4/CCL5 and CXCR3- CXCL9/CXCL10/CXCL11/CXCL13 axis were significantly correlated with CD 4 T and CD 8 T cells, indicating that these interactions may be essential for the recruitment of the T lymphocytes into tumor. These findings may provide a new clue for biomarkers in melanoma.

A recent study reported four molecular subtypes for melanoma that were proven to be beneficial for highlighting key potential subtype-specific drug targets (9). The proportions of the four TME subtypes in each molecular subtype were comparable. Next, we combined the genes used for molecular subtyping with the identified significantly mutated genes, and investigated their distributions in the immune subtypes. The two driver genes, CDKN2A and PRB2, had significantly different MAFs between the high-immunity and low-immunity. In terms of the CNVs, CDKN2A and PTEN were significantly deleted in the low-immunity cohort, whereas RAC1 was significantly amplified in the low-immunity cohort. These observations suggested that these mutations may be the potential biomarkers to predict clinical benefits in melanoma, and enable the reasonable selection of anti-melanoma treatments. Our study will provide a deep understanding of melanoma immune microenvironment and molecular characteristics, and facilitate the development of new therapeutic strategies for melanoma patients. There are some limitations of our study. The candidate biomarkers were achieved by computational methods. Experiments are needed to verify their roles in the clinical therapy of melanoma. Also, the biomarkers were identified based on the limited melanoma samples, and a larger cohort is needed to reveal biological signatures to improve the anti-melanoma strategies.

## Data Availability Statement

The public datasets of TCGA-SKCM, phs001036, phs000452 and SRP070710 for this study can be found in the Cancer Genome Atlas (TCGA, https://portal.gdc.cancer.gov/) and SRA databases.

## Author Contributions

TJ, JM and XC conceived the idea, designed the research concept of this manuscript, JW conducted the collection of data and did the analyses and drafted the manuscript. JM, CP, and WD helped in analysis. TJ and JM revised the manuscript. All authors read and approved the final manuscript.

## Funding

This work was supported by the National Natural Science Foundation of China (32070678, 32000472); the Emergency Key Program of Guangzhou Laboratory, Grant No. EKPG21-12; the National Key Research and Development Program of China(2020YFC0840800); CAMS Innovation Fund for Medical Sciences(CIFMS)(2021-I2M-1-061).

## Conflict of Interest

The authors declare that the research was conducted in the absence of any commercial or financial relationships that could be construed as a potential conflict of interest.

## Publisher’s Note

All claims expressed in this article are solely those of the authors and do not necessarily represent those of their affiliated organizations, or those of the publisher, the editors and the reviewers. Any product that may be evaluated in this article, or claim that may be made by its manufacturer, is not guaranteed or endorsed by the publisher.

## References

[1] Xiao D Barry S Kmetz D Egger M Pan J Rai SN. Melanoma Cell-Derived Exosomes Promote Epithelial-Mesenchymal Transition in Primary Melanocytes Through Paracrine/Autocrine Signaling in the Tumor Microenvironment. Cancer Lett (2016) 376(2):318–27. doi: 10.1016/j.canlet.2016.03.050 PMC4869527 27063098

[2] Sung H Ferlay J Siegel RL Laversanne M Soerjomataram I Jemal A. Global Cancer Statistics 2020: GLOBOCAN Estimates of Incidence and Mortality Worldwide for 36 Cancers in 185 Countries. CA Cancer J Clin (2021) 71(3):209–49. doi: 10.3322/caac.21660 33538338

[3] Marzagalli M Ebelt ND Manuel ER. Unraveling the Crosstalk Between Melanoma and Immune Cells in the Tumor Microenvironment. Semin Cancer Biol (2019) 59:236–50. doi: 10.1016/j.semcancer.2019.08.002 31404607

[4] Liu D Yang X Wu X. Tumor Immune Microenvironment Characterization Identifies Prognosis and Immunotherapy-Related Gene Signatures in Melanoma. Front Immunol (2021) 12:663495. doi: 10.3389/fimmu.2021.663495 34025664 PMC8134682

[5] Binnewies M Roberts EW Kersten K Chan V Fearon DF Merad M. Understanding the Tumor Immune Microenvironment (TIME) for Effective Therapy. Nat Med (2018) 24(5):541–50. doi: 10.1038/s41591-018-0014-x PMC5998822 29686425

[6] Jacquelot N Duong CPM Belz GT Zitvogel L. Targeting Chemokines and Chemokine Receptors in Melanoma and Other Cancers. Front Immunol (2018) 9:2480. doi: 10.3389/fimmu.2018.02480 30420855 PMC6215820

[7] Thorsson V Gibbs DL Brown SD Wolf D Bortone DS Ou Yang TH. The Immune Landscape of Cancer. Immunity (2018) 48(4):812–30. e14. doi: 10.1016/j.immuni.2018.03.023 29628290 PMC5982584

[8] Bagaev A Kotlov N Nomie K Svekolkin V Gafurov A Isaeva O. Conserved Pan-Cancer Microenvironment Subtypes Predict Response to Immunotherapy. Cancer Cell (2021) 39(6):845–65.e7. doi: 10.1016/j.ccell.2021.04.014 34019806

[9] Cancer Genome Atlas N. Genomic Classification of Cutaneous Melanoma. Cell (2015) 161(7):1681–96. doi: 10.1016/j.cell.2015.05.044 PMC4580370 26091043

[10] Hayward NK Wilmott JS Waddell N Johansson PA Field MA Nones K. Whole-Genome Landscapes of Major Melanoma Subtypes. Nature (2017) 545(7653):175–80. doi: 10.1038/nature22071 28467829

[11] Liang WS Hendricks W Kiefer J Schmidt J Sekar S Carpten J. Integrated Genomic Analyses Reveal Frequent TERT Aberrations in Acral Melanoma. Genome Res (2017) 27(4):524–32. doi: 10.1101/gr.213348.116 PMC5378171 28373299

[12] Van Allen EM Miao D Schilling B Shukla SA Blank C Zimmer L. Genomic Correlates of Response to CTLA-4 Blockade in Metastatic Melanoma. Science (2015) 350(6257):207–11. doi: 10.1126/science.aad0095 PMC5054517 26359337

[13] Hugo W Zaretsky JM Sun L Song C Moreno BH Hu-Lieskovan S. Genomic and Transcriptomic Features of Response to Anti-PD-1 Therapy in Metastatic Melanoma. Cell (2016) 165(1):35–44. doi: 10.1016/j.cell.2016.02.065 26997480 PMC4808437

[14] Dobin A Davis CA Schlesinger F Drenkow J Zaleski C Jha S. STAR: Ultrafast Universal RNA-Seq Aligner. Bioinformatics (2013) 29(1):15–21. doi: 10.1093/bioinformatics/bts635 23104886 PMC3530905

[15] Li B Dewey CN. RSEM: Accurate Transcript Quantification From RNA-Seq Data With or Without a Reference Genome. BMC Bioinf (2011) 12:323. doi: 10.1186/1471-2105-12-323 PMC3163565 21816040

[16] Zhang Y Parmigiani G Johnson WE. ComBat-Seq: Batch Effect Adjustment for RNA-Seq Count Data. NAR Genom Bioinform (2020) 2(3):lqaa078. doi: 10.1093/nargab/lqaa078 33015620 PMC7518324

[17] Mermel CH Schumacher SE Hill B Meyerson ML Beroukhim R Getz G. GISTIC2.0 Facilitates Sensitive and Confident Localization of the Targets of Focal Somatic Copy-Number Alteration in Human Cancers. Genome Biol (2011) 12(4):R41. doi: 10.1186/gb-2011-12-4-r41 21527027 PMC3218867

[18] Yoshihara K Shahmoradgoli M Martinez E Vegesna R Kim H Torres-Garcia W. Inferring Tumour Purity and Stromal and Immune Cell Admixture From Expression Data. Nat Commun (2013) 4:2612. doi: 10.1038/ncomms3612 24113773 PMC3826632

[19] Miao YR Zhang Q Lei Q Luo M Xie GY Wang H. ImmuCellAI: A Unique Method for Comprehensive T-Cell Subsets Abundance Prediction and Its Application in Cancer Immunotherapy. Adv Sci (Weinh) (2020) 7(7):1902880. doi: 10.1002/advs.201902880 32274301 PMC7141005

[20] Lawrence MS Stojanov P Polak P Kryukov GV Cibulskis K Sivachenko A. Mutational Heterogeneity in Cancer and the Search for New Cancer-Associated Genes. Nature (2013) 499(7457):214–8. doi: 10.1038/nature12213 PMC3919509 23770567

[21] Mayakonda A Lin DC Assenov Y Plass C Koeffler HP. Maftools: Efficient and Comprehensive Analysis of Somatic Variants in Cancer. Genome Res (2018) 28(11):1747–56. doi: 10.1101/gr.239244.118 PMC6211645 30341162

[22] Gu Z Eils R Schlesner M. Complex Heatmaps Reveal Patterns and Correlations in Multidimensional Genomic Data. Bioinformatics (2016) 32(18):2847–9. doi: 10.1093/bioinformatics/btw313 27207943

[23] Love MI Huber W Anders S. Moderated Estimation of Fold Change and Dispersion for RNA-Seq Data With Deseq2. Genome Biol (2014) 15(12):550. doi: 10.1186/s13059-014-0550-8 25516281 PMC4302049

[24] Yu G Wang LG Han Y He QY. Clusterprofiler: An R Package for Comparing Biological Themes Among Gene Clusters. OMICS (2012) 16(5):284–7. doi: 10.1089/omi.2011.0118 PMC3339379 22455463

[25] Wu T Hu E Xu S Chen M Guo P Dai Z. Clusterprofiler 4.0: A Universal Enrichment Tool for Interpreting Omics Data. Innovation (N Y) (2021) 2(3):100141. doi: 10.1016/j.xinn.2021.100141 34557778 PMC8454663

[26] Andrews LP Marciscano AE Drake CG Vignali DA. LAG3 (CD223) as a Cancer Immunotherapy Target. Immunol Rev (2017) 276(1):80–96. doi: 10.1111/imr.12519 28258692 PMC5338468

[27] Gebauer F Kramer M Bruns C Schlosser HA Thelen M Lohneis P. Lymphocyte Activation Gene-3 (LAG3) mRNA and Protein Expression on Tumour Infiltrating Lymphocytes (TILs) in Oesophageal Adenocarcinoma. J Cancer Res Clin Oncol (2020) 146(9):2319–27. doi: 10.1007/s00432-020-03295-7 PMC7382658 32592066

[28] Shi AP Tang XY Xiong YL Zheng KF Liu YJ Shi XG. Immune Checkpoint LAG3 and Its Ligand FGL1 in Cancer. Front Immunol (2021) 12:785091. doi: 10.3389/fimmu.2021.785091 35111155 PMC8801495

[29] Young A Ngiow SF Gao Y Patch AM Barkauskas DS Messaoudene M. A2AR Adenosine Signaling Suppresses Natural Killer Cell Maturation in the Tumor Microenvironment. Cancer Res (2018) 78(4):1003–16. doi: 10.1158/0008-5472.CAN-17-2826 29229601

[30] Hajaj E Eisenberg G Klein S Frankenburg S Merims S Ben David I. SLAMF6 Deficiency Augments Tumor Killing and Skews Toward an Effector Phenotype Revealing It as a Novel T Cell Checkpoint. Elife (2020) 9:1-23. doi: 10.7554/eLife.52539 PMC7075692 32122464

[31] Manieri NA Chiang EY Grogan JL. TIGIT: A Key Inhibitor of the Cancer Immunity Cycle. Trends Immunol (2017) 38(1):20–8. doi: 10.1016/j.it.2016.10.002 27793572

[32] Netanely D Leibou S Parikh R Stern N Vaknine H Brenner R. Classification of Node-Positive Melanomas Into Prognostic Subgroups Using Keratin, Immune, and Melanogenesis Expression Patterns. Oncogene (2021) 40(10):1792–805. doi: 10.1038/s41388-021-01665-0 PMC7946641 33564068

[33] Martins WK Esteves GH Almeida OM Rezze GG Landman G Marques SM. Gene Network Analyses Point to the Importance of Human Tissue Kallikreins in Melanoma Progression. BMC Med Genomics (2011) 4:76. doi: 10.1186/1755-8794-4-76 22032772 PMC3212933

[34] Li B Severson E Pignon JC Zhao H Li T Novak J. Comprehensive Analyses of Tumor Immunity: Implications for Cancer Immunotherapy. Genome Biol (2016) 17(1):174. doi: 10.1186/s13059-016-1028-7 27549193 PMC4993001

[35] Johrer K Pleyer L Olivier A Maizner E Zelle-Rieser C Greil R. Tumour-Immune Cell Interactions Modulated by Chemokines. Expert Opin Biol Ther (2008) 8(3):269–90. doi: 10.1517/14712598.8.3.269 18294099

[36] Franciszkiewicz K Boissonnas A Boutet M Combadiere C Mami-Chouaib F. Role of Chemokines and Chemokine Receptors in Shaping the Effector Phase of the Antitumor Immune Response. Cancer Res (2012) 72(24):6325–32. doi: 10.1158/0008-5472.CAN-12-2027 23222302

[37] Cirenajwis H Lauss M Ekedahl H Torngren T Kvist A Saal LH. NF1-Mutated Melanoma Tumors Harbor Distinct Clinical and Biological Characteristics. Mol Oncol (2017) 11(4):438–51. doi: 10.1002/1878-0261.12050 PMC5527484 28267273

[38] Han Y Li X Yan J Ma C Wang X Pan H. Bioinformatic Analysis Identifies Potential Key Genes in the Pathogenesis of Melanoma. Front Oncol (2020) 10:581985. doi: 10.3389/fonc.2020.581985 33178610 PMC7596746

[39] Liu H Yang Z Lu W Chen Z Chen L Han S. Chemokines and Chemokine Receptors: A New Strategy for Breast Cancer Therapy. Cancer Med (2020) 9(11):3786–99. doi: 10.1002/cam4.3014 PMC7286460 32253815

