# Supplementary material for: Exploring Tumor Immune Microenvironment and Its Associations With Molecular Characteristics in Melanoma
Source: Front Oncol. 2022 Apr 21;12:821578. doi: 10.3389/fonc.2022.821578 (PMC9069107; doi:10.3389/fonc.2022.821578)
Supplement: Supplementary file 1 [file DataSheet_1.docx]

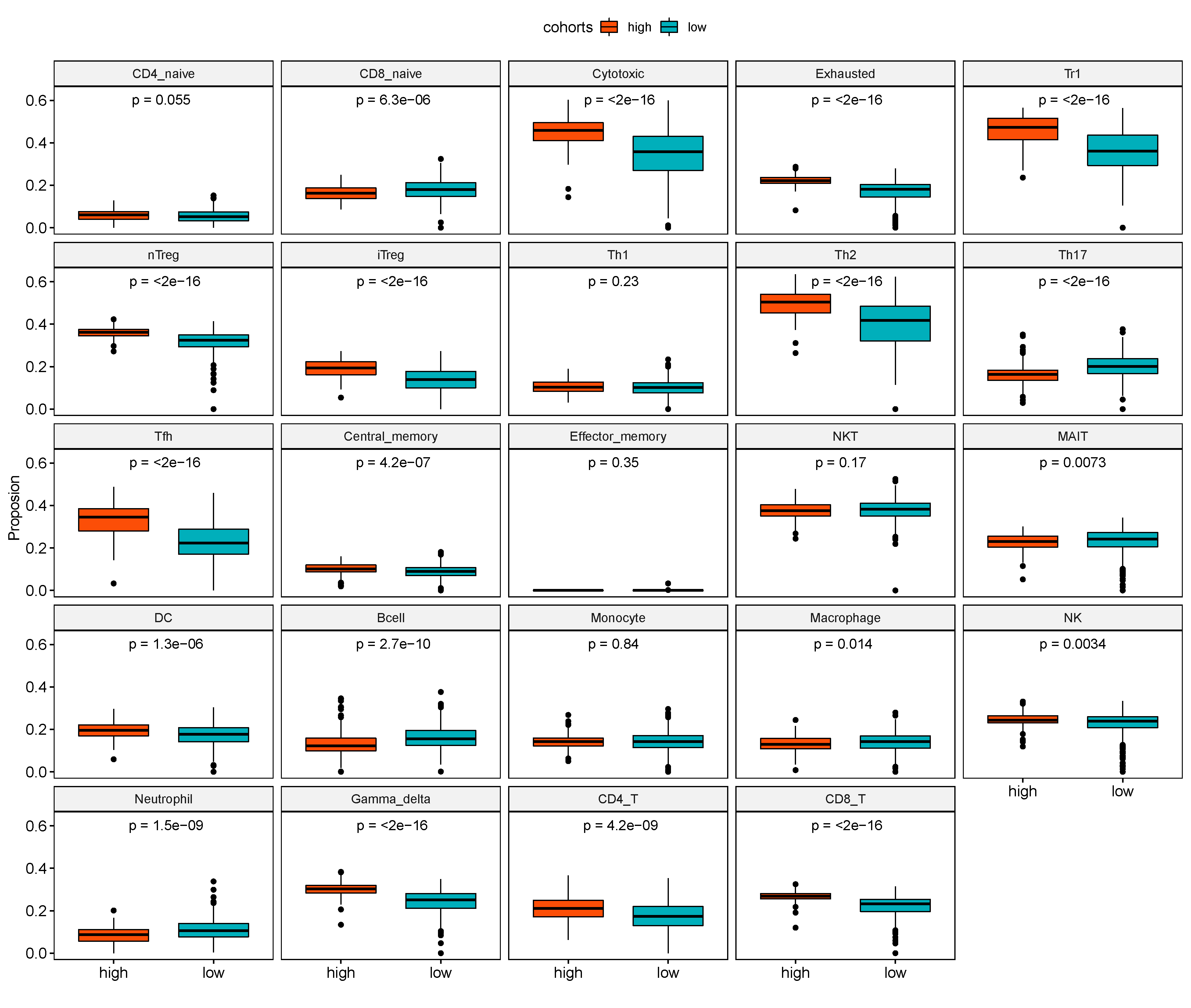


Figure S1. The compositions of 24 immune cells varied between the high-immunity and low-immunity cohorts (Mann-Whitney U test).


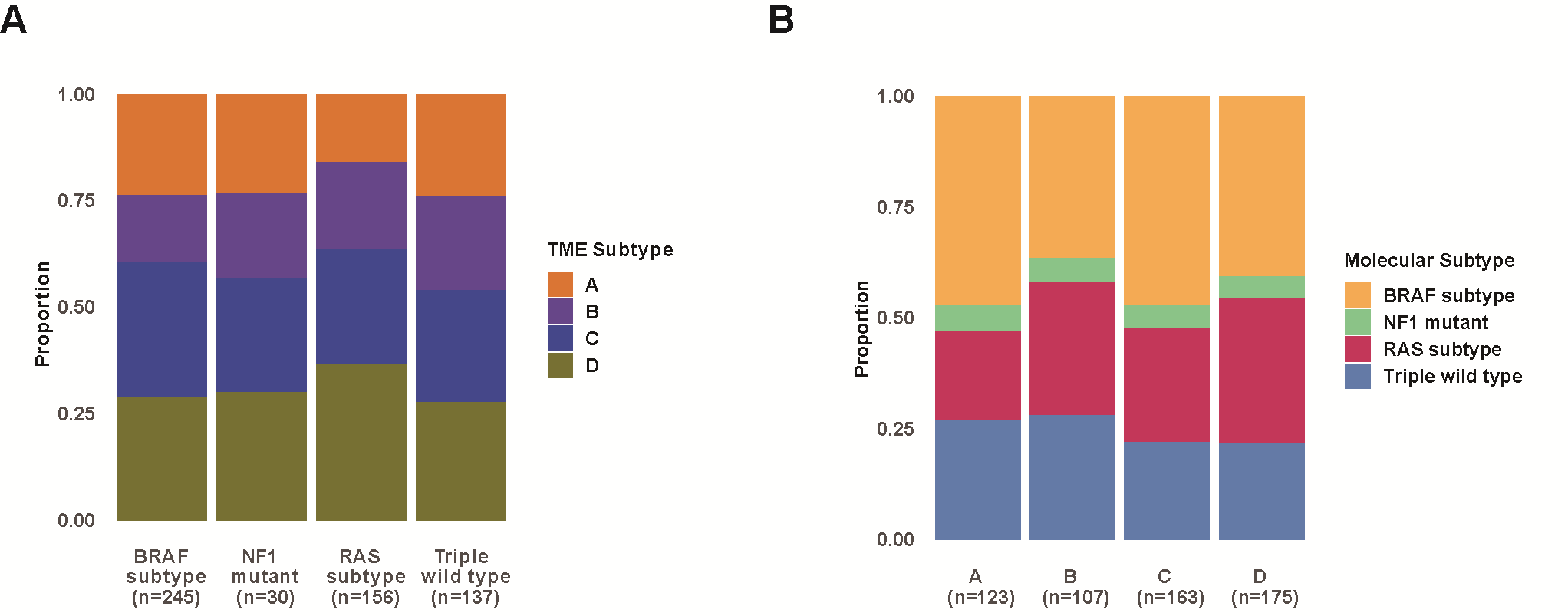


Figure S2. The correlation between molecular subtypes and TME subtypes. (A) The proportions of the four TME subtypes in each molecular subtype. (B) The proportions of the four molecular subtypes in each TME subtype.


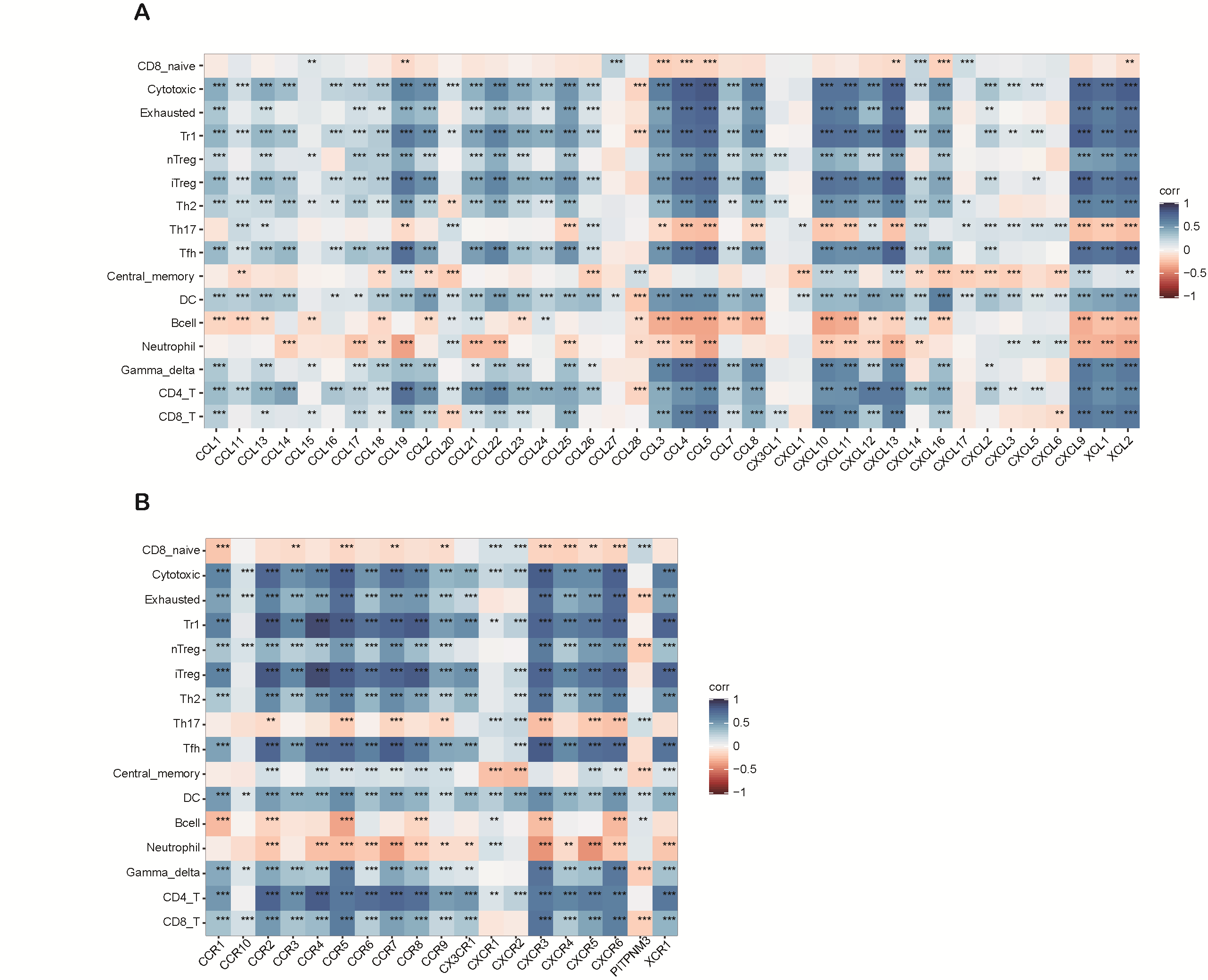


Figure S3. The correlations of chemokines and receptors with immune cell infiltration. (A) The correlations between chemokines and immune cell infiltration. * p < 0.05; ** p<0.01;*** p<0.001. (B) The correlations between chemokine receptors and immune cell infiltration. * p < 0.05; ** p<0.01;*** p<0.001.
